# Dietitians’ Perspectives on the Coordination and Continuity of Nutrition Care for Malnourished or Frail Clients: A Qualitative Study

**DOI:** 10.3390/healthcare10060986

**Published:** 2022-05-26

**Authors:** Megan Rattray, Shelley Roberts

**Affiliations:** 1 School of Health Sciences and Social Work, Griffith University, Gold Coast 4222, Australia; s.roberts@griffith.edu.au; 2 College of Medicine & Public Health, Flinders University, Adelaide 5042, Australia; 3 Allied Health Research, Gold Coast Hospital and Health Service, Gold Coast 4215, Australia

**Keywords:** community, frailty, malnutrition, nutrition care, transitions in care

## Abstract

Malnutrition and frailty are common conditions that impact overall health and function. There is limited research exploring the barriers and enablers to providing coordinated nutrition care to malnourished or frail clients in the community (including transitions from hospital). This study aimed to explore dietitians’ experiences and perspectives on providing coordinated nutrition care for frail and malnourished clients identified in the community or being discharged from hospital. Semi-structured interviews with clinical/acute, community, and aged care dietitians across Australia and New Zealand were conducted. Interviews were 23–61 min long, audio recorded and transcribed verbatim. Data were analysed using inductive thematic analysis. Eighteen dietitians participated in interviews, including five clinical, eleven community, and two residential aged care dietitians. Three themes, describing key factors influencing the transition and coordination of nutrition care, emerged from the analysis: (i) referral and discharge planning practices, processes, and quality; (ii) dynamics and functions within the multidisciplinary team; and (iii) availability of community nutrition services. Guidelines advising on referral pathways for malnourished/frail clients, improved communication between acute and community dietitians and within the multidisciplinary team, and solutions for community dietetic resource shortages are required to improve the delivery of coordinated nutrition care to at-risk clients.

## 1. Introduction

Malnutrition and frailty are significant problems for both hospitalised and community-dwelling adults. Around 1–17% of community-dwelling adults are malnourished and 4–63% are at risk of malnutrition in developed countries such as Australia and New Zealand [1]. One-third of hospital inpatients are also estimated to be malnourished [2], many of whom are discharged back into the community without resolution of malnutrition [3]. This results in poor outcomes for patients (higher infection and complication rates [4], increased falls [5], impaired wound healing [6], longer length of hospital stay, and increased morbidity and mortality [7]) and health services (increased costs, higher readmission rates [4,7]). Similarly, frailty, which can be described as a loss of physiological reserve leading to diminished adaptive capacity and adverse outcomes [8,9], is highly prevalent among community-dwelling adults, with 2–29% estimated to be frail and 41- 54% estimated to be prefrail in developed countries [1]. Nutrition is considered a key component in frailty concepts [10] and while these two conditions are distinct from each other, they share some characteristics, causes/risk factors, and outcomes [11]. As such, they are often treated simultaneously, particularly in elderly persons [12].

Poor energy and protein intake and diet quality are major modifiable risk factors in the development and progression of both malnutrition and frailty [1,10]. Consequently, nutrition-based strategies aiming to influence an individual’s knowledge and behaviour around dietary intake are often employed to treat these conditions. In fact, providing nutrition care to malnourished or frail clients after hospital discharge has been shown to reduce avoidable readmissions, improve intake and weight gain [13,14], ameliorate gait speed [15], and reduce 6-month and 90-day mortality by 7.8% [16] and 4.9% [17], respectively. Despite this, many adults who need nutrition care in this transition period do not receive it [18]. Further, many community-dwelling adults with or at risk of malnutrition or frailty are poorly recognised and hence do not receive the nutrition care they need [18]. These findings demonstrate that nutrition care for adults who are frail, malnourished, or at risk of developing these conditions during the hospital-to-home transition period and in the community needs improvement.

While previous work has described communication problems between health care professionals (HCPs) and across health care services, and insufficient knowledge/attention to nutritional needs/problems by HCPs as barriers to the continuity of nutrition care [19,20,21], these findings have been generated from studies exclusively conducted in clinical settings or studies that have solely focused on transitions of care between acute and community settings. Further, few studies have explored dietitians’ perceptions of the barriers and enablers to providing coordinated care to malnourished and frail clients in the community. Given interviewing key stakeholders allows researchers to understand a phenomenon of interest on a deeper level [22], the aim of the present study was to explore key factors influencing coordinated and continuous nutrition care for frail or malnourished clients who are identified in the community or who are transitioning home from the hospital, through interviewing accredited practicing dietitians. The findings of this research will provide insight into the strategies required for effective coordination and transitions of nutrition care to improve outcomes for malnourished or frail clients.

## 2. Materials and Methods

### 2.1. Study Design

This descriptive qualitative study was part of a larger project involving the development of an evidence- and data-informed guide for managing malnutrition and frailty in the Australian and New Zealand (ANZ) community setting. Individual semi-structured interviews were conducted to explore dietitians’ views on the coordination and transition of nutrition care for clients who are malnourished or frail in ANZ communities. This design was appropriate as it provides a deeper understanding of the phenomenon of interest and allows researchers to understand the world from the participants’ perspective [22]. This study received ethical approval from the participating university (Griffith University Human Research Ethics Committee: 2021/048). The methodology and reporting of this study were devised according to the Consolidated Criteria for Reporting Qualitative Research (COREQ) [23].

### 2.2. Setting and Sample

Dietitians were recruited through Part A of our project, a national survey assessing dietitians’ perspectives on providing nutrition support and advice to malnourished or frail clients in the community (reported elsewhere). Dietitians were eligible to participate in the survey, and thus to be interviewed, if they were a part- or full-time clinical/acute, community/primary care or aged care dietitian currently practicing in Australia or New Zealand, and provided care to clients who were malnourished, frail, or at risk. A question was embedded at the end of the survey asking participants if they were interested in being contacted for a semi-structured interview to explore their perceptions regarding the topics of malnutrition/frailty in the community in greater depth, with a brief description of what their participation would involve. Dietitians who expressed interest (n = 31) were sent an information sheet via email by a member of the study team (MR) and a mutually acceptable time for the interview was arranged. Verbal consent was reconfirmed with each participant at the beginning of the interview. Participants were purposefully selected from the pool of those who expressed interest, to ensure maximum variation in relation to state, region, area of practice, and years of experience. It was estimated that around 15–20 dietitians would be interviewed based on studies with a similar design [24]; however, recruitment continued until data saturation was reached (i.e., when no more new information could be gathered) [25].

### 2.3. Data Collection

Interviews were conducted between April and June 2021. A semi-structured interview guide, underpinned by Van Houdt et al.’s care coordination framework [26], was used to explore participants’ experiences. One pilot interview was conducted to: (i) check that the questions were successful in drawing out the information being sought, (ii) assess the wording clarity and identify any redundant questions, and (iii) ascertain the correct order of questions to make the interview flow logically. A member of the research team (MR) conducted all interviews one-on-one with participants over the phone or via videoconferencing, at a date and time convenient to them. Interviews lasted between 23 and 61 min (average 41 min), were audio recorded and then transcribed verbatim for analysis. Examples of the interview questions under each domain of the semi-structured guide are shown in Table 1. 

### 2.4. Data Analysis

Data were analysed using inductive thematic analysis following Braun and Clarke’s six-step guide [27] to identify emerging themes. This involved one author (MR) reading and re-reading transcripts for data immersion; developing codes based on participants’ verbatim statements; grouping codes into themes according to similarity; generating a ‘thematic map’; defining and naming themes; and writing up findings with supporting participant quotes. An electronic audit trail was reviewed by another researcher (SR) from the coding step onwards. The analysis was an iterative process, with the researchers having regular discussions and referring back to the raw data to substantiate emerging ideas and themes. The labelling of themes/subthemes was discussed among both researchers until consensus was reached.

To maximise trustworthiness and limit threats to validity, several strategies outlined by Lincoln and Guba [28] were employed. For example, we present detailed and in-depth descriptive data and include rich verbatim quotes from participants that depict each theme to meet the criteria for transferability and confirmability, respectively. Dependability was enacted by maintaining memos throughout data analysis to document the analytical decisions made. Lastly, we enacted the criterion for credibility through open-ended questioning, spending a prolonged amount of time with the material and giving a detailed description of the methods.

## 3. Results

### 3.1. Demographics

Eighteen participants, including five clinical, eleven community, and two residential aged care dietitians, were interviewed. The participant demographics are outlined in Table 2. All participants were female and had been practicing for a median (IQR) of 10.5 (6–19) years. Most participants delivered nutrition therapy in Australia (n = 13) and in a metropolitan area (n = 11) at the time interviews took place. Participants from all but one territory/state in Australia were interviewed while there was representation from five regions across New Zealand.

Participant responses formed three themes and various subthemes, described in detail below. Key factors influencing the transition and coordination of nutrition care for malnourished and frail clients in Australia and New Zealand were identified throughout these themes.

### 3.2. Theme 1: Referral and Discharge Planning Practices, Processes, and Quality

Timely, appropriate, and comprehensive referrals were identified as a key step in the transition and coordination of nutrition care. Factors that appeared to influence referral processes included: (i) the requestor’s background and training, (ii) the type of systems and programs in operation, and (iii) whether guidelines or directives were established for discharge planning/referrals to community or residential aged care dietitians. 

#### 3.2.1. Requestor’s Background and Training Influence Referral Quality

All community and residential aged care dietitians described receiving discharge plans/referrals for frailty and malnutrition from various requestors, including clinical dietitians, general practitioners (GPs), allied health professionals, nurses, rest homes, and service providers (e.g., My Aged Care). However, perceptions around the quality and appropriateness of referrals varied depending on who the referral was coming from. Generally, referrals received from clinical dietitians were described as “good” or “excellent”, as they were appropriate, described what nutrition care had been provided in hospital, and included the client’s detailed medical and social history. This information was important to community and residential aged care dietitians to triage incoming referrals and provide tailored and timely care to clients. Referrals from service providers, intrahospital transfers, and GPs were commonly described as “poor”, often being attributed to inadequate information being provided such as the reason for referral. This was particularly problematic for dietitians who did not have access to clients’ electronic medical records or have open lines of communication with the requestor to confirm or request additional information.

*“We get direct referrals from the hospital dietitians mostly. But also from like doctors and clinics and things, or nurse clinics as well. And we also accept referrals through My Aged Care… Certain dietitians give a lot more information in their referral than others… If we get a referral from My Aged Care, it’s very limited information, because a lot of the time, it might not be done by someone who’s necessarily a health person… and then doctors, they tend to give us…a medical history and medication but sometimes not a lot on actual what’s happening with their nutrition, it might just be, ‘please review’. So it definitely depends on who it’s coming from.”*

(P06, Community Dietitian, Australia)

#### 3.2.2. Systems and Programs Influence Referral Intake

How community and residential aged care dietitians received referrals varied greatly and were frequently cited as a barrier or enabler to the provision of nutrition care. Often those who spoke highly of the discharge planning/referral process were in a workplace where the referral system was electronic, integrated, and open lines of communication existed between requestors and recipients. These were community dietitians who worked within specialised programs (e.g., Transitional Aged Care Program, Commonwealth Health Support Program), where resources had been allocated to screening and referring, and referrals were generally received from a single port of entry. As a result, referrals were informative, appropriate, and received in a timely manner. Alternatively, dietitians who worked in part-time and/or public health community positions held mixed views on how well the referral pathway operated in their place of work. Often the referral process in these areas were described as “ad hoc” and “fragmented”. One community dietitian stated that she received more referrals from staff outside her organisation, which she attributed to the poor exchange of information systems at her workplace, while another described the referral process being heavily reliant on clinicians knowing each other. The latter was perceived to be a problem when strong working relationships had not been formed between the community dietitian and other HCPs working in the community and/or clinical setting. 

#### 3.2.3. Protocols, Guidelines, or Work Instructions can Guide Practice

Most dietitians were not aware of any protocols, guidelines, or work instructions to advise on transitions or coordination of care in their place of work. However, this was not the case for all dietitians. Several community dietitians who worked within specialised programs stated that they had work instructions to guide their referral/intake processes and advise on practice, which appeared to positively influence the intake and coordination of care. Regardless of whether protocols, guidelines, or work instructions were in existence at participants’ place of work, most dietitians were in favour of having guidance around the management of malnutrition and frailty in the community being introduced for all HCPs, to standardise practice and reduce ambiguity, particularly for referring and managing clients when they are discharged home. This was seen as important by many dietitians, who thought more could be done for malnourished and/or frail clients in the community, and that a guide would draw attention to this matter.

*“If there were clear guidelines on how patients should be managed once they’re discharged from hospital, who is the best placed person to see them or clearer recommendations about handover of care—I would definitely use that. I think at the moment part of my personal problem is that I don’t think it is clear.”*

(P01, Clinical dietitian, Australia)

### 3.3. Theme 2: Dynamics and Functions within the Multidisciplinary Team

Dynamics and functions within the multidisciplinary team were identified as key factors in the transition and coordination of nutrition care. Factors that appeared to influence team dynamics and functions included: (i) roles and responsibilities, (ii) extent and form of communication, and (iii) engagement approaches.

#### 3.3.1. Divergence in Expected and Actual Roles within the Team

Each HCP’s role in the transition and coordination of nutrition care was discussed by dietitians. It was unanimously agreed that everyone involved in the care of a malnourished and/or frail client should be aware and involved in helping them reach their nutritional goals. Participants outlined that the role of a community dietitian in the transition of care process should involve continuing the nutrition support put in place in the hospital, while reviewing/adapting the client’s needs/goals to ensure a smooth tarnation home. Providing timely care and tailored education were also identified by some dietitians, along with liaising with the client’s GP to provide a holistic approach. In addition to dietitians, GPs and community nurses were the most cited HCPs that should be involved, given their consistent interactions with clients and because some health districts were reliant on GPs to refer to a dietitian. However, while ideal roles were consistently described, mixed remarks were provided around the extent to which such responsibilities were undertaken in different work settings. For example, while GPs were widely considered to have central roles, many dietitians spoke of them not being as involved as they potentially could be in the context of screening, diagnosing, and follow-up. Further, it was acknowledged that while acute dietitians should provide a comprehensive nutrition-related handover to either the facility the client is being discharged to or the community dietitian, it is not considered standard practice in all workplaces.

*“I think they (GPs) do have a huge role to play… we don’t have enough dietitians in the workforce or the funding to enable dietitians to be doing it (screening), so we need those in primary care to be doing the screening—that would be a great place for it to happen. And then based on the outcomes of screening, referring on for further, you know, diagnosis and treatment as needed… But I think more community education would be really good.”*

(P10, Community dietitian, New Zealand)

#### 3.3.2. Extent and Form of Communication Varies

The extent and form of communication with HCPs, regardless of the discipline, was frequently cited as a barrier/enabler regarding the transition and coordination of care. For example, several community dietitians said they engaged in regular in-person contact with other HCPs, including clinical dietitians, which they perceived to strengthen their working relationship, resulting in the exchange and handover of more comprehensive and efficient client information. This was usually enabled through continuing staff education sessions and shared multidisciplinary office spaces. Alternatively, when community dietitians had not formed close working relationships or were unknown to other HCPs, communication was often described as “ad hoc” and “non-existent”. This impacted negatively on client care as referrals were less frequent and handovers were poor. Further, communication with GPs and occasionally nurses was often described as “one-way” by both clinical and community dietitians, with communication most frequently occurring via documentation. One clinical dietitian spoke of her frustration with not receiving confirmation following the referral of her malnourished or frail clients to GPs once discharged from hospital:

*“I would feel like I was able to close the loop much more efficiently and I would be getting better patient care if I could do that, to know the referral has happened and the patient is in good hands versus when you do it to a GP, you just don’t know what’s going to become of it, whether it’s in the too hard basket.”*

(P08, Clinical Dietitian, Australia)

While it was widely acknowledged that GPs “should always be kept in the loop”, one community dietitian outright stated she would never “tell” a client’s GP they were malnourished given she did not have a clear line of communication, and she anticipated an underwhelming response such as “oh, ok”.

#### 3.3.3. HCP Engagement Dependent on Various Factors

HCP engagement, to some extent, appeared to be indirectly influenced by whether community dietitians worked within specialised community programs and/or were affiliated/had arrangements with local hospitals/services. Indeed, those working within specialised programs consistently described referral quality, follow-up, and exchange of information with other HCPs as “good”, attributing this to strong working relationships and acknowledgment of the importance of nutrition within their teams. However, engagement also appeared to be directly and positively influenced by proactive attempts by community dietitians to engage with HCPs within their team and/or service(s) and educate them on malnutrition and frailty and what their role is in the community in the context of these issues. This was often achieved through in-services, ‘road shows’, and visits to the GPs. When such activities were not undertaken, particularly by dietitians in public health community positions, it appeared to hinder their working relationships and in turn their referral rate and quality. In fact, one clinical dietitian stated she did not know who the community dietitians were in her area, which she attributed to their absence of self-promotion. While continued HCP education and self-promotion were consistently raised as important factors for building relationships and gaining buy-in, one community dietitian outlined the consistent challenges encountered when engaging with and educating GP practices:

*“GP practices can be challenging for us because we want them to be educated more than they want to be educated by us, so it’s a bit of a push. We always reach out to them really; I think we see the need for it more so. But we usually try to do a bit of an in-service of, you know, we’ll try and pick like a focus area. As much as overall, we’re usually just trying to encourage referrals… we’ll usually do it over their lunch break because that’s the only time you can actually get them as a collective group… They’re usually obliging enough, but we could probably do a bit more of it sometimes to.”*

(P13, Community Dietitian, Australia)

### 3.4. Theme 3: Availability of Community Nutrition Services

The availability and accessibility of (i) primary care dietitians and (ii) community services was identified as a key factor influencing the transition and coordination of nutrition care.

#### 3.4.1. Community Resources and Funding Shortages

A shortage of nutrition professionals and services in the community was identified as a gap in the transitional care of frail/malnourished clients between the hospital and home. In fact, around one-third of participants listed a lack of community dietitians and/or nutrition services as a primary barrier in the transition and provision of care among frail and/or malnourished clients. These participants included a mix of clinical and community dietitians from metropolitan and regional regions across Australia. Contrastingly, one clinical dietitian working in a regional area thought there were enough dietitians in the community but that there were insufficient services in lower socio-economic districts. Long waitlists were seen as a consequence of insufficient staffing and/or service and the negative impact this had on client care was acknowledged by all:

*“There’s a lack of community dietetic services available to us. We’re underserviced in that area I would say, which does impact on patient care… we don’t really have that service (community dietitian), so patients who are going to be followed up as an outpatient have a really long waiting period because we’ve just got so many patients on the waitlist and not enough, I guess dietitians to service that area.”*

(P01, Clinical Dietitian, Australia)

#### 3.4.2. Extending Services in Acute Settings to Facilitate Care

The clinical dietitians spoke about how they currently navigate the shortage of community dietitians and services. For some, this involved providing an extension of care from the hospital. This occurred in the form of providing clients with a small storage of free or subsidised ONS to use at home and/or checking in on them once they were home. Two participants shared that they would occasionally refer their patients to a GP in the hope that they would be linked in with a private practice dietitian, who might be able to see them in a timelier manner. While others thought direct follow-up with clients outside of the clinical setting was outside their scope of practice and indicated they did not know who was available to handover to:

*“Sometimes we do [provide an extension of care from hospital], but it’s fairly rare... I feel like once they’re discharged from hospital, there’s no one to hand over to. The care stops with them discharging. So sometimes, I call the GP practices to talk to the practice nurse or someone about handover.”*

(P09, Clinical dietitian, Australia)

## 4. Discussion

This study explored dietitians’ perceptions on providing coordinated nutrition care for frail and malnourished patients being discharged from hospital or identified in the community. Referral processes and quality, dynamics and functions within the multidisciplinary team, and the availability of community nutrition services were identified as key factors influencing the coordination of nutrition care. These findings can be used to inform guidance for multidisciplinary HCPs on identifying and managing malnutrition and frailty in the community to improve outcomes for community-dwelling clients.

Effective referral practices and discharge planning could significantly improve a client’s health and reduce their risk of admission/readmission to hospital [29]. However, as demonstrated within the current study, receiving timely, appropriate, and comprehensive referrals in primary care is problematic, leading to delays in the delivery of nutrition care to clients. In view of the importance of timely and comprehensive referrals, many countries have recently launched guidelines to advise on screening, referral, and management practices for nutrition care in tertiary and primary care contexts [30,31]. Indeed, previous work has reported that nutrition pathways for interdisciplinary care resulted in practice improvements [32,33] and were favourably viewed as a solution to poor care transitions in the current study. However, a challenge in developing such pathways is that primary health models and community services vary across countries and regions. Consequently, organisations need to adapt these pathways to their specific context, considering the community resources available (e.g., services and community dietitians). For example, acute and community organisations should collaboratively adapt pre-established referral and discharge planning guidelines for nutrition care, based on their specific context, to facilitate timely and appropriate referrals for malnourished/frail clients. Templates and instructions outlining what information needs to be included should accompany any referral guideline to facilitate comprehensive information transfer to community and aged care dietitians to base client care off, which was perceived as important in the current study. 

The coordination and management of malnutrition and frailty care should involve collaboration within the multidisciplinary team, as suggested by the current study and previous work [34]. Of particular importance is the collaborative effort between dietitians and GPs to triage clients and assist them in achieving their nutrition goals. However, dietitians occupying public community nutrition positions in the current study indicated the overwhelming hardship in engaging with GPs, other community HCPs, and, at times, acute care dietitians. Previous work has reported that GPs feel they do not know who is responsible for the management of malnutrition and frailty in the community setting and expressed their need for more support from other HCPs to effectively monitor and treat these conditions [35,36,37]. Further, it has been suggested that staff in acute and community settings need opportunities to gain a better understanding of each other’s roles and build relationships and trust [38], as each discipline has vastly different ideas and opinions on community-based care management [39]. This suggests there is a clear need for dietitians in primary care to engage in efforts to educate and support staff to understand their role, and to deliver high-quality care to patients. Face-to-face interactions, such as delivering presentations and multidisciplinary team meetings, can promote collaborative practice and mutual understanding between various professions, as suggested by previous work [39] and participants in the current study. Further, an understanding of each discipline’s and practitioner’s communication preferences is required. A review found that GPs generally prefer short written communications that include goals and plans that are easily identified in a structured report (e.g., assessment, diagnosis, intervention, monitoring, and evaluation) [40]; these findings appear consistent with the current study, given this was the most frequent form of communication with GPs as reported by dietitians. 

Limited availability and accessibility of community dietitians and services was identified as a key barrier, a finding congruent with previous work [30,41,42]. Some acute dietitians within the current study spoke about navigating this pitfall by providing an extension of care to their clients (i.e., following up with clients following discharge and/or providing them with ONS to use at home). This may be an effective solution to counteract community resource shortages given an exploratory analysis found that most at-risk/malnourished/frail clients followed information provided in-hospital about food intake and community food-related services post-discharge [43]. Further, a recent quality improvement initiative that aimed to introduce a dietitian-led discharge planning and follow-up program demonstrated that it was feasible to integrate a post-discharge role into acute dietetic practice [15]. Lastly, upskilling non-dietetic HCPs in the community to provide nutritional intervention when a dietitian is unavailable has been shown to be effective at improving outcomes in malnourished clients [44], and is consequently outlined in best practice guidelines [31]. Given simply increasing community nutrition resources to meet demand is likely not an option for most regions, finding a workaround such as those suggested above is likely to improve the coordination and management of nutrition care for community-dwelling malnourished or frail clients.

This study has several strengths and limitations. A strength of this study is the generalisability of our findings, given participants from two countries and across different geographical regions were interviewed. Further, we were able to provide practical recommendations based on our findings, which, if addressed, could enhance the coordination of nutrition care provided to clients who are malnourished and frail (Box 1). Limitations include the potential that not all views were captured; however, we mitigated this by using purposive sampling and continuing data collection until saturation was reached.

Box 1 **Implications and recommendations for practice.**

- Acute and community organisations should collaboratively adapt pre-established referral and discharge planning guidelines for nutrition care, based on their specific context, to facilitate referrals and discharge planning by providing guidance on the process and content.
- Opportunities should be provided for community-based dietitians to build relationships with and educate other HCPs (including acute care dietitians) on dietitians’ role in managing malnutrition and frailty in the community, to assist HCPs to work in a more integrated way.
- Acute care organisations should consider providing extended nutrition care to frail/malnourished clients after discharge to improve client outcomes, given the shortage of community dietitians and services available in some regions.
- Non-dietetic HCPs in the community should be upskilled to screen for malnutrition and frailty and provide nutritional support to malnourished/frail clients when a dietitian is unavailable.

## 5. Conclusions

This study focused specifically on the factors influencing care transitions and the coordination of nutrition care in the community for malnourished and frail clients. From the experiences reported, it is clear there is a need for guidelines advising on referral pathways for at-risk/malnourished/frail clients; improved communication between acute and community dietitians and among the multidisciplinary team; and solutions for community dietetic resource shortages, such as training non-dietetic HCPs to provide nutritional management and/or for acute organisations to provide extended care to discharged malnourished/frail clients. Future work should consider assessing the effectiveness of and processes underpinning a program of research implementing these recommendations. 

## Figures and Tables

**Table 1:** Semi-structured interview guide.

Concept	Specific Concepts	Questions
(Inter) organisational mechanisms	External factors, structure, tasks characteristics	Do you currently have any polices or guidelines at your place of work to advise on the transition of care of malnutrition/frailty in the community? How has the coordination of care and management of malnutrition/frailty in the community changed over the past 10 years (or since you arrived in your current workplace)?
	Cultural factors, knowledge, and technology	Are there any enablers or barriers to providing nutrition care to frail/malnourished patients in the transition from hospital to home/in the community? Are there any specific documents, materials or resources you currently use to guide your practice when managing frail and/or malnourished patients? What do you like or dislike about these materials/documents? Do they provide sufficient guidance for managing frail/malnourished patients after hospital discharge/at home in the community?
	Need for coordination, administrative operational processes	Tell me about your sense of the need for coordination between the hospital and community for patients who or malnourished or frail? How is/should this exchange of information occur?
Relational coordination	Quality of relationship, exchange of information	Can you tell me about the relationship between the healthcare professionals in the hospital and community setting?
	Roles, goals	What role do clinical (acute) dietitians play in the transition of malnourished patients from hospital to home? What role do community dietitians play in the transition of malnourished patients from hospital to home? What role do general practitioners play in the transition of malnourished patients from hospital to home and in the community? What role do community nurses play in the transition of malnourished patients from hospital to home and in the community? What role do other allied health professionals play in the transition of malnourished patients from hospital to home and in the community?
Outcomes	Team, patient, and/or (inter) organizational outcome	Do you think improving the coordination of malnourished and/or frail patients in the community could lead to positive impacts? Do you think improving the delivering of nutrition care to malnourished and/or frail patients in the community could lead to positive impacts?

**Table 2:** Demographic information of participants (n = 18).

Characteristic	n (%)
Years practicing	
1–5	3 (17%)
6–10	6 (33%)
11–15	4 (22%)
16–20	1 (6%)
>21	4 (22%)
Country	
Australia	13 (72%)
New Zealand	5 (28%)
Region	
Metropolitan	11 (61%)
Regional	4 (22%)
Rural or remote	3 (17%)
Area of practice	
Clinical	5 (28%)
Community/Primary Care	11 (61%)
Residential aged care	2 (11%)

## Data Availability

Data will be made available upon reasonable request to authors.
